# Solasonine Modulates Aldose Reductase Activity in *Galleria mellonella* (Lepidoptera): Implications for the Polyol Pathway

**DOI:** 10.3390/toxins18100410

**Published:** 2026-09-23

**Authors:** Marta Spochacz-Santoro, Szymon Chowański, Karolina Walkowiak-Nowicka, Monika Szymczak-Cendlak, Zbigniew Adamski

**Affiliations:** 1 Department of Animal Physiology and Developmental Biology, Institute of Experimental Biology, Faculty of Biology, Adam Mickiewicz University, 61-614 Poznan, Poland; szymon.chowanski@amu.edu.pl (S.C.); karolina.walkowiak@amu.edu.pl (K.W.-N.); monika.szymczak@amu.edu.pl (M.S.-C.); zbigniew.adamski@amu.edu.pl (Z.A.); 2 Institute of Human Biology and Evolution, Faculty of Biology, Adam Mickiewicz University, 61-614 Poznan, Poland; 3 Laboratory of Electron and Confocal Microscopy, Faculty of Biology, Adam Mickiewicz University, 61-614 Poznan, Poland

**Keywords:** solasonine, aldose reductase, polyols, hemolymph, fat body, *Galleria mellonella*

## Abstract

Glycoalkaloids are plant secondary metabolites known to affect multiple physiological processes in insects; however, their effects on carbohydrate and polyol metabolism remain poorly understood. This study investigated the effects of solasonine on hemolymph metabolites and aldose reductase activity in the fat body of *Galleria mellonella* larvae. Larvae were fed a diet supplemented with five nominal dietary concentrations of solasonine (10^−4^–1 mg/kg diet) for three days. Hemolymph metabolites were analyzed by RP-HPLC, and aldose reductase activity was determined in fat body lysates. Inositol levels differed significantly among experimental groups (one-way ANOVA, *p* < 0.0001), with the 10^−4^ mg/kg diet treatment differing significantly from the vehicle control (*p* = 0.0015). Aldose reductase activity was significantly lower at 10^−2^, 10^−3^, and 10^−4^ mg/kg diet compared with the untreated control (*p* = 0.0439, *p* = 0.027, and *p* < 0.0001, respectively), while only the 10^−4^ mg/kg treatment differed significantly from the vehicle control (*p* = 0.0029). Formic acid also significantly affected several metabolic parameters. Overall, solasonine exposure was associated with altered aldose reductase activity and changes in polyol-related metabolites, although some metabolic effects may be attributable to the vehicle.

## 1. Introduction

Glycoalkaloids are secondary metabolites produced by plants, particularly those belonging to the Solanaceae family, where they serve as part of the plant’s chemical defense against herbivores. Their content varies considerably depending on the plant species and tissue, developmental stage, environmental conditions, and other factors. The biological activity of glycoalkaloids depends on both the steroidal aglycone and the attached carbohydrate moiety. Solasonine, one of the major glycoalkaloids found in several *Solanum* species, consists of the steroidal alkaloid aglycone solasodine linked to a trisaccharide chain composed of galactose, mannose, and glucose. This structure gives the molecule amphiphilic properties, with a relatively hydrophobic steroidal domain and a hydrophilic carbohydrate moiety. Such structural features are important for its interaction with biological membranes. Steroidal glycoalkaloids can interact with membrane sterols, including cholesterol, and disturb membrane integrity and permeability, with the extent of these effects depending on the structure of both the aglycone and carbohydrate moiety [1]. These membrane disturbances can subsequently affect cellular homeostasis and contribute to toxic effects. Glycoalkaloids have attracted considerable attention because of their insecticidal and sublethal effects. Both purified compounds and plant extracts containing glycoalkaloids have been investigated in a range of insect species [2]. Their effects are not restricted to mortality but may also involve changes in development, reproduction, nervous system function, membrane integrity, and metabolism. For example, α-solanine and α-tomatine affected the retrocerebral complex and reproduction-related structures in *Tenebrio molitor* and *Zophobas atratus*, with the magnitude of the response depending on both the compound and insect species [3]. Glycoalkaloids have also been shown to interfere with energy metabolism. In *T. molitor*, solanine, chaconine, tomatine, and tomato leaf extract altered the activity and expression of enzymes involved in glycolysis, the Krebs cycle, and fatty acid β-oxidation, demonstrating that their effects extend beyond direct membrane damage [4]. Other recent studies have reported insecticidal activity of *Solanum*-derived preparations against important pests. For example, *Solanum incanum* leaf powder showed insecticidal activity against *Sitophilus zeamais*, while *Solanum virginianum* extracts showed larvicidal activity against *Spodoptera frugiperda* and were investigated for their potential effects on juvenile hormone esterase [5,6]. Recent studies have also demonstrated the insecticidal potential of *Solanum* extracts against *Galleria mellonella*, with an ethanol extract of *Solanum elaeagnifolium* seeds causing concentration-dependent larval mortality and an LC_50_ of 198.3 µg/mL [7]. These findings further support the potential of Solanaceae-derived compounds as sources of botanical insecticides. Despite the well-documented biological activity of glycoalkaloids, their effects on insect metabolism are still not fully understood. Importantly, the response to a particular glycoalkaloid may differ substantially between insect species and may also depend on the tissue examined, exposure conditions, and compound concentration. Therefore, further studies are needed to identify metabolic processes that may be affected by glycoalkaloid exposure.

One metabolic process that may be relevant to the response to glycoalkaloids is the polyol pathway. In insects, aldose reductase can catalyze the NADPH-dependent reduction of glucose to sorbitol, which can subsequently be converted to fructose by sorbitol dehydrogenase [8,9]. The production of polyols may contribute to osmotic regulation and adaptation to environmental or metabolic stress. Aldose reductase belongs to the larger aldo-keto reductase (AKR) superfamily [10], whose members use NADPH as a reducing cofactor and catalyze the reduction of various carbonyl-containing compounds, including aldehydes, ketones, monosaccharides, ketosteroids, and prostaglandins [11]. Consequently, AKR enzymes may participate in processes extending beyond carbohydrate metabolism, including the metabolism of endogenous carbonyl compounds and the detoxification of reactive or xenobiotic metabolites. AKR enzymes have been identified in several insect tissues, although their distribution and function are dependent on the species and individual enzyme. For example, AKR2E8 has been characterized in the silkworm *Bombyx mori*. Its transcript was detected in the testes, ovaries, and fat body, with the highest expression observed in the midgut, and the recombinant enzyme was shown to reduce several aldehydes using NADPH [12]. These findings indicate that insect AKR enzymes may be involved in both metabolism and detoxification [13]. The fat body is particularly relevant in this context because it is a major metabolic and storage organ in insects, involved in the regulation of carbohydrate, lipid, and protein metabolism and in the release of nutrients and metabolites into the hemolymph. Changes in NADPH-dependent reductase activity in this tissue could therefore be associated with broader metabolic responses to chemical stress. The rationale for investigating this process in *G. mellonella* comes from our earlier observations. We previously demonstrated that exposure of *G. mellonella* larvae to *Solanum nigrum* extract and its major glycoalkaloid, solasonine, altered the concentrations of several hemolymph metabolites, including glucose, trehalose, sorbitol, mannitol, erythritol, inositol, and proline [14]. In particular, changes in both sugars and polyols suggested that glycoalkaloid exposure may affect carbohydrate and polyol metabolism. However, the enzymatic mechanisms underlying these changes have not yet been investigated. This provided the basis for examining whether exposure to solasonine is associated with changes in aldose reductase activity in the fat body, together with changes in the hemolymph metabolite profile. *G. mellonella* is also a relevant species for studying the physiological effects of plant-derived defensive compounds. In addition to being a widely used laboratory model in physiological, toxicological, and microbiological research, the greater wax moth is an economically important pest of honey bee colonies [15]. Its larvae feed on wax and other components of honeycomb and can cause considerable damage to managed bee colonies. Recent research has highlighted the need for effective and sustainable approaches to its control [16]. Furthermore, *G. mellonella* may be exposed to residues of pesticides used in beekeeping, and recent studies have shown that such exposure can affect detoxification-related processes, metabolism, and gene expression in the fat body and midgut [17,18]. Thus, this species provides a useful model for studying how exposure to plant-derived or synthetic xenobiotics affects insect metabolism and detoxification. The hemolymph provides an important link between metabolically active tissues and serves as a medium for the transport of nutrients and metabolic products. In *G. mellonella*, trehalose is a major circulating carbohydrate, while glucose, fructose, amino acids such as proline, and several sugar alcohols can also be detected in the hemolymph [14,19,20]. Circulating carbohydrates provide important energy substrates, whereas polyols such as sorbitol and mannitol may additionally contribute to osmotic regulation and cellular protection. The fat body plays a central role in maintaining this metabolic balance by storing nutrients and regulating their mobilization and release into the hemolymph. Therefore, examining aldose reductase activity in the fat body together with changes in circulating sugars and polyols may provide a better understanding of the metabolic response of *G. mellonella* to solasonine exposure. In the present study, we investigated whether exposure of *G. mellonella* larvae to different concentrations of solasonine alters the hemolymph metabolite profile and aldose reductase activity in fat body lysates. Hemolymph and fat body samples were collected from treated larvae, and changes in selected hemolymph metabolites were evaluated together with aldose reductase activity in the fat body. By combining these measurements, we aimed to determine whether changes in aldose reductase activity are associated with the metabolic alterations observed following solasonine exposure.

## 2. Results

### 2.1. Mortality

Among the control groups, mean mortality (±SD) ranged from 3.3 ± 7.0% to 6.6 ± 8.7%. In the solasonine-treated groups, mean mortality was 15.0 ± 18.5% at 10^−4^ mg/kg diet, 6.6 ± 9.4% at 10^−3^ mg/kg diet, and 0% at 10^−2^, 0.1, and 1 mg/kg diet. Overall, mortality remained low and showed substantial variability between replicates, with no statistically significant differences between treatment groups. Thus, the mortality data did not indicate a significant dose-dependent effect of solasonine under the experimental conditions.

### 2.2. Hemolymph Metabolite Profiling

RP-HPLC (Reversed-phase High-Performance Liquid Chromatography) analysis of the hemolymph samples revealed multiple peaks corresponding to metabolites present in both control and treated groups. Quantitative analysis revealed statistically significant differences in metabolite levels across the tested solasonine concentrations compared to the control or vehicle control (Figure 1). All raw results obtained from RP-HPLC are available in Supplementary Data.

#### 2.2.1. Polyols

The hemolymph levels of inositol, sorbitol, erythritol, and mannitol, as well as total polyol levels, were determined following solasonine exposure. Inositol levels differed significantly among the experimental groups (one-way ANOVA, *p* < 0.0001; FDR-adjusted *p* < 0.0009). The vehicle control showed markedly higher inositol levels than the untreated control (1.682 ± 0.3124 vs. 0.1413 ± 0.1068 µg/µL, respectively; *p* < 0.0001). Among the solasonine treatments, only the 10^−4^ mg/kg diet treatment differed significantly from the vehicle control, with inositol levels decreasing from 1.682 ± 0.3124 to 1.168 ± 0.1578 µg/µL, corresponding to a 30.6% decrease (*p* = 0.0015).

Sorbitol was detected at very low levels and was not detectable in most samples. The mean concentration was 0.0510 ± 0.0572 µg/µL in the untreated control, 0.00517 ± 0.00574 µg/µL at 10^−4^ mg/kg diet, and 0 µg/µL in the vehicle control and at 10^−3^ and 10^−2^ mg/kg diet. Although the Kruskal–Wallis test indicated an overall difference among the experimental groups (*p* = 0.0348), this difference did not remain significant after FDR correction (FDR-adjusted *p* = 0.1044), and Dunn’s multiple-comparison test revealed no significant pairwise differences.

Erythritol levels also showed an overall difference among the experimental groups (one-way ANOVA, *p* = 0.0325); however, this difference was not significant after FDR correction (FDR-adjusted *p* = 0.1044). The only significant pairwise difference was observed between the untreated and vehicle controls, with erythritol levels increasing from 0.6382 ± 0.3237 to 1.025 ± 0.1800 µg/µL, corresponding to a 60.6% increase (*p* = 0.0437). No significant differences were observed between the solasonine treatments and either control. No significant differences were observed in mannitol levels among the experimental groups (one-way ANOVA, *p* = 0.3329).

Total polyol levels were significantly higher in the vehicle control than in the untreated control (6.247 ± 1.116 vs. 3.858 ± 1.408 µg/µL, respectively), corresponding to a 61.9% increase (*p* = 0.0157). Total polyol levels were also significantly higher in the 10^−3^ and 10^−2^ mg/kg diet solasonine treatments than in the untreated control, by 76.0% (6.792 ± 1.260 µg/µL; *p* = 0.0024) and 32.2% (5.098 ± 1.017 µg/µL; *p* = 0.0086), respectively. However, because the vehicle control itself significantly differed from the untreated control, these differences cannot be attributed exclusively to solasonine exposure. The overall difference in total polyol levels remained significant after FDR correction (FDR-adjusted *p* = 0.0103).

#### 2.2.2. Sugars

No significant differences were observed in hemolymph trehalose levels among the experimental groups (one-way ANOVA, *p* = 0.4315; FDR-adjusted *p* = 0.471). Similarly, saccharose levels did not differ significantly among the groups (one-way ANOVA, *p* = 0.6486; FDR-adjusted *p* = 0.6486). For glucose, significant heterogeneity of variances was detected by the Brown–Forsythe (*p* = 0.0010) and Bartlett’s (*p* < 0.0001) tests. Therefore, Welch’s ANOVA was used, which showed no significant differences in glucose levels among the experimental groups (W(4, 10.97) = 1.622, *p* = 0.2375; FDR-adjusted *p* = 0.381). Mean glucose concentrations were 0.5443 ± 0.2317 µg/µL in the untreated control, 0.6838 ± 0.4791 µg/µL in the vehicle control, 0.3657 ± 0.0953 µg/µL at 10^−4^ mg/kg diet, 1.314 ± 1.424 µg/µL at 10^−3^ mg/kg diet, and 0.4152 ± 0.4226 µg/µL at 10^−2^ mg/kg diet.

#### 2.2.3. Amino Acids

The hemolymph levels of proline and valine did not differ significantly among the experimental groups. Proline concentrations ranged from 5.657 ± 0.9916 to 6.845 ± 0.9814 µg/µL, with no significant differences detected among groups (one-way ANOVA, *p* = 0.3452; FDR-adjusted *p* = 0.414). Similarly, no significant differences were observed in valine levels (one-way ANOVA, *p* = 0.1954; FDR-adjusted *p* = 0.381), with mean concentrations ranging from 0.4148 ± 0.0978 to 0.6335 ± 0.1197 µg/µL. A numerical downward trend was observed in total amino acid levels with increasing solasonine concentration. Mean concentrations decreased from 7.269 ± 1.041 µg/µL at 10^−4^ mg/kg diet to 6.940 ± 1.364 µg/µL at 10^−3^ mg/kg diet and 6.072 ± 0.9966 µg/µL at 10^−2^ mg/kg diet, corresponding to a 16.5% decrease between the lowest and highest solasonine concentrations. However, the overall difference among the experimental groups was not statistically significant (one-way ANOVA, *p* = 0.2537; FDR-adjusted *p* = 0.381).

### 2.3. Aldose Reductase Activity

Aldose reductase activity in fat body samples differed significantly among the experimental groups according to Welch’s one-way ANOVA (F(6, 26.54) = 20.19, *p* < 0.0001; Figure 2). Mean aldose reductase activity was 179.5 mU/mg protein in the untreated control and 127.4 mU/mg protein in the vehicle control, corresponding to a 29.0% decrease in the vehicle group. Games–Howell multiple-comparisons analysis showed that aldose reductase activity at 10^−4^ mg/kg diet (33.0 mU/mg protein) was 81.6% lower than in the untreated control (*p* < 0.0001) and 74.1% lower than in the vehicle control (*p* < 0.0001). The activity at 10^−4^ mg/kg diet was also significantly lower than that observed at 10^−3^, 10^−2^, 0.1, and 1 mg/kg diet, by 66.1%, 69.6%, 74.3%, and 80.7%, respectively (*p* = 0.0191, *p* = 0.0224, *p* = 0.0021, and *p* = 0.0021). No significant differences were observed among the higher solasonine concentrations or between these treatments and either control group. Overall, the results indicate an association between solasonine exposure and altered aldose reductase activity, with the most pronounced difference observed at the lowest nominal dietary concentration tested (10^−4^ mg/kg diet). All raw data for aldose reductase activity are provided in the Supplementary Data.

## 3. Discussion

### 3.1. Aldose Reductase Activity and Potential Metabolic Implications

Aldo-keto reductases (AKRs) constitute a broad family of NADPH-dependent enzymes involved in the reduction of endogenous carbonyl compounds, xenobiotics, and other chemically reactive substrates. In insects, AKR enzymes have been identified in metabolically active tissues, including the midgut and fat body, although their tissue distribution and substrate specificity are isoform- and species-dependent. The fat body was selected in the present study because it is a major metabolic and detoxification tissue in insects. AKR enzymes have previously been detected in the fat body of lepidopteran species, including *Bombyx mori*, where individual AKR isoforms have been shown to exhibit NADPH-dependent reductase activity and detoxification-related functions [9,12]. However, the assay used in the present study measures aldose reductase activity based on NADPH consumption and does not establish the molecular identity of the enzyme responsible. Therefore, the observed changes should be interpreted as alterations in aldose reductase activity rather than as evidence for modulation or inhibition of a specific AKR isoform.

The most pronounced difference in aldose reductase activity was observed after application of the lowest nominal dietary concentration of solasonine (10^−4^ mg/kg diet). Importantly, this treatment differed significantly from both the untreated and vehicle controls, whereas the other solasonine concentrations did not differ significantly from either control. Thus, the present results provide evidence for an association between exposure to the lowest tested concentration of solasonine and altered aldose reductase activity, but they do not support a general concentration-dependent inhibitory effect of solasonine on this enzyme activity. The non-monotonic pattern observed across the tested concentrations should likewise be interpreted cautiously. A formal dose–response analysis with a larger number of concentrations and biological replicates would be required to test this possibility.

Aldose reductase is classically associated with the first step of the polyol pathway, in which glucose is reduced to sorbitol. The pathway is also involved in glucose sensing and metabolic regulation in insects, suggesting that changes in aldose reductase activity could potentially be associated with alterations in carbohydrate and polyol metabolism. However, the relationship between enzyme activity and individual polyol concentrations is not necessarily direct. Sorbitol and other polyols can be generated through multiple metabolic routes, and their concentrations are additionally affected by substrate availability, subsequent metabolism, transport, and excretion. Therefore, the reduced aldose reductase activity observed at a concentration of 10^−4^ mg/kg diet cannot by itself be interpreted as inhibition of the entire polyol pathway. Similarly, the present experiments do not establish that changes in polyol concentrations result directly from altered aldose reductase activity.

This interpretation is particularly relevant when the present findings are considered in the context of previous studies on glycoalkaloid toxicity in insects. Steroidal glycoalkaloids can interact with biological membranes and alter membrane integrity and permeability, while studies in insects have also reported changes in metabolism and stress-related processes following exposure to glycoalkaloids [4,21]. Such effects may result from several interconnected physiological responses rather than from a single metabolic pathway. This interpretation is consistent with recent findings showing that exposure of *G. mellonella* to the beehive acaricide tau-fluvalinate can affect growth as well as carbohydrate and lipid metabolism [18]. The observed alteration in aldose reductase activity may therefore represent one component of a broader metabolic response to solasonine exposure. Nevertheless, oxidative stress was not directly assessed in the present study, and therefore a direct relationship between aldose reductase activity and oxidative stress cannot be established.

### 3.2. Hemolymph Metabolites

Changes in hemolymph metabolites provided additional evidence of metabolic responses following exposure to solasonine; however, the effects should be interpreted in relation to the vehicle control. Sorbitol levels showed a significant overall difference among the experimental groups according to the Kruskal–Wallis test, but no individual pairwise comparison was statistically significant. Moreover, the vehicle control showed a metabolic profile that differed from the untreated control for several parameters. Therefore, the observed variation in sorbitol concentrations cannot be attributed specifically to solasonine exposure. These findings illustrate the importance of including an appropriate vehicle control when evaluating metabolic responses to poorly water-soluble compounds.

Inositol and erythritol also differed between the untreated and vehicle controls, indicating that formic acid contributed to some of the observed metabolic variation. In contrast, the significant difference in inositol between the 10^−4^ mg/kg diet treatment and the vehicle control suggest that this parameter may also be responsive to solasonine exposure under the experimental conditions used. However, because the vehicle itself produced substantial metabolic effects, the biological interpretation of these changes should remain cautious.

Total polyol levels were higher in the vehicle control than in the untreated control and were also significantly higher in some solasonine-treated groups relative to the untreated control. However, the significant effect of the vehicle indicates that these changes cannot be attributed exclusively to solasonine. After correction for multiple testing, total polyol levels remained significantly different among the experimental groups, supporting the presence of an overall metabolic effect, but this statistical result does not identify solasonine as the sole factor responsible for the observed variation. The present findings therefore support an association between treatment conditions and polyol-related metabolism rather than demonstrating direct activation or inhibition of the polyol pathway.

Glucose concentrations did not differ significantly among the experimental groups when the unequal variances were taken into account using Welch’s ANOVA. Although greater variability was observed at some solasonine concentrations, increased dispersion alone does not demonstrate disruption of metabolic homeostasis. The variability may reflect differences in individual physiological responses, but this remains a possible explanation rather than a demonstrated mechanism. Similarly, no significant differences were observed for trehalose, saccharose, proline, valine, or total amino acid levels. The absence of statistically significant changes in these parameters suggests that the metabolic response to the tested exposure conditions was not uniform across all carbohydrate and amino acid pools.

### 3.3. Role of the Vehicle Study and Limitations

Formic acid was used to facilitate solasonine administration because of its limited solubility in water. The present results demonstrate that the vehicle itself affected several hemolymph metabolic parameters and therefore represents an important experimental consideration. Formic acid is biologically active and is used in apiculture for the control of *Varroa destructor* [22], further supporting the need to consider its potential effects on arthropod physiology. However, the present study was not designed to investigate the physiological effects of formic acid, and the observed metabolic changes should therefore not be extrapolated to a broader toxicological effect of the compound. Importantly, the fact that some parameters differed between solasonine treatments and the vehicle control indicates that solasonine may still contribute to specific responses, but these effects should be distinguished from vehicle-associated changes.

Several limitations should be considered when interpreting the present findings. First, the number of biological replicates was relatively limited, which reduces statistical power and may contribute to variability in the measured responses. Although each hemolymph sample was obtained by pooling material from five larvae, the pooled samples represented the biological replicates for statistical analysis. Second, measurements were performed at a single time point (3 days after exposure). Consequently, the temporal relationship between changes in aldose reductase activity and hemolymph metabolites cannot be determined. A time-course experiment would be necessary to establish whether changes in enzyme activity precede, accompany, or follow alterations in polyol concentrations. Third, the enzymatic assay does not identify the molecular isoform responsible for the measured activity. Direct biochemical experiments using purified recombinant enzymes, together with molecular identification and expression analysis of candidate AKR genes, would be required to determine the specific enzyme involved. Finally, the present study did not directly measure oxidative stress, polyol flux, glucose-to-sorbitol conversion, or xenobiotic elimination. Therefore, the proposed links between aldose reductase activity, polyol metabolism, oxidative stress, and detoxification should be considered hypotheses for future investigation rather than established mechanisms.

Overall, the present findings indicate that solasonine exposure is associated with an alteration in aldose reductase activity in the fat body of *G. mellonella*, most clearly at the lowest nominal dietary concentration tested, together with changes in selected hemolymph metabolites. However, the metabolic effects of the vehicle emphasize the importance of vehicle-controlled comparisons. The results are consistent with the possibility that aldose reductase activity forms part of the metabolic response to glycoalkaloid exposure, but they do not establish direct inhibition of a specific AKR isoform or inhibition of the polyol pathway as a whole. Further studies combining molecular identification of AKR enzymes, direct enzyme–compound assays, time-course experiments, and measurements of polyol flux and oxidative stress will be necessary to clarify the mechanisms underlying the observed responses.

## 4. Conclusions

The present study indicates that solasonine exposure was associated with altered aldose reductase activity in the fat body of *G. mellonella*, with the most pronounced difference observed at the lowest nominal dietary concentration tested. Changes in selected hemolymph metabolites, particularly polyol-related metabolites, were also observed, although some effects were associated with the vehicle. Nevertheless, these findings provide a basis for further investigation of how steroidal glycoalkaloids influence insect metabolism and whether changes in aldose reductase activity represent a primary enzymatic response or a secondary consequence of broader metabolic alterations.

## 5. Materials and Methods

### 5.1. Insect Treatment

Fourth-instar (L4) larvae of *G. mellonella* were selected from the breeding colony and placed in Falcon tubes, with 5–6 individuals per tube. Each treatment group consisted of at least two Falcon tubes. The experiments were independently repeated at least three times (biological replicates), resulting in a minimum of 30 larvae per concentration. The experiments were conducted under controlled conditions (29 ± 1 °C, 70 ± 5% relative humidity, and complete darkness), as in the breeding colony. Larvae were fed for three days with an artificial diet consisting of homogenized oat flakes and honey mixed at a 1:1 ratio. The diet was supplemented with 100 µL of an α-solasonine solution (Sigma-Aldrich, St. Louis, MO, USA). Pure solasonine was initially dissolved in 1% formic acid to facilitate complete dissolution of the glycoalkaloid, and subsequent dilutions were prepared using water. Aliquots of 100 µL of solasonine solutions containing 0.1, 10^−2^, 10^−3^, 10^−4^, or 10^−5^ mg of solasonine were thoroughly mixed with 10 g of diet. This resulted in nominal dietary concentrations of 1, 0.1, 10^−2^, 10^−3^, and 10^−4^ mg/kg diet, respectively. For the hemolymph metabolite analysis, three solasonine concentrations (10^−2^, 10^−3^, and 10^−4^ mg/kg diet) were used, whereas five concentrations (1, 0.1, 10^−2^, 10^−3^, and 10^−4^ mg/kg diet) were used for the aldose reductase activity assay. The broader concentration range was used for the latter experiment to provide a more comprehensive assessment of the response to solasonine exposure. Control groups received the same diet supplemented with 100 µL of water or water containing 0.001% formic acid. The latter control corresponded to the maximum amount of formic acid introduced into the diet with the highest solasonine treatment. Larval mortality was assessed after three days in both preliminary experiments conducted before the main experiments and in the subsequent main experimental assays. Each experiment was independently repeated at least three times, with a minimum of two Falcon tubes per treatment group in each experiment. Mortality was calculated for each concentration, and differences between treatment groups were analyzed using the Kruskal–Wallis test followed by Dunn’s multiple-comparison test. Across all preliminary and main experiments, a total of 548 larvae were included in the mortality assessment.

### 5.2. Hemolymph and Fat Body Tissues Collection

After three days of treatment, the mortality of larvae was assessed. Larvae were anaesthetized with carbon dioxide, and one leg from the third pair of thoracic (true) legs, counting from the anterior end of the larva, was amputated. To each sample, 10 µL of hemolymph was collected with an automatic pipette (2 µL from each individual) and pooled in 600 µL of 70% ethanol. The larvae were cut with scissors, and fat body tissue was collected into Eppendorf tubes (Eppendorf, Hamburg, Germany) kept on ice to avoid enzyme activity and protein degradation. For each concentration, at least 8 samples of the fat body were collected with at least 40 individuals per group, and for hemolymph at least 6 samples per concentration with at least 30 individuals in each group.

### 5.3. RP-HPLC Assay

Samples were shaken and centrifuged for 10 min at 10,000× *g*, filtered with Millex SamplicityTM filters with pore size 0.20 µm (Merck Millipore, Darmstadt, Germany), and analyzed using RP-HPLC. For separation, a DionexUltimate 3000 chromatographic system comprising a dual-pump programmable solvent module and a Corona Charged Aerosol Detector (CAD) (Thermo Fisher Scientific, Waltham, MA, USA) was used, as described previously by [14]). Briefly, the samples were analysed with an Asahipak NH2P-50 4E column (250 × 4.6 mm, Shodex, Tokyo, Japan) and eluted by multi-step gradient of ACN concentration and flow rate as follows: 0–5 min (86%, 1 mL/min), 5–10 min (83%, 1 mL/min), 10–20 min (81%, 1 mL/min), 20–38 min (81%, 1.4 mL/min), and 38–40 min (86%, 1 mL/min) at 38 °C. As standards, we used substances of the analyzed compounds (Merck, Darmstadt, Germany) as solutions in 70% ethanol at a concentration of 1 mg/mL.

### 5.4. Aldose Reductase Activity Assay

The activity of aldose reductase in samples of fat body tissue was analyzed using a colorimetric assay kit (ab273276, Abcam, Cambridge, UK), according to the manufacturer’s instructions as follows. The samples of fat body tissue (50–100 mg) were isolated into 300 µL of ice-cold AR Assay Buffer containing 10 µM DTT, kept on ice for 10 min, and finally homogenized with a pestle hand homogenizer. The procedure was done on ice to avoid protein degradation. Next, the lysates were centrifuged at 12,000× *g* at 4 °C for 10 min. The resulting supernatant was transferred into fresh tubes, and 2 μL aliquots were collected at this stage for the determination of soluble protein concentration. Protein concentration was determined using a Direct Detect infrared spectrometer (Merck), following the method described by Szymczak-Cendlak et al. [23]. The protein content of the analysed tissues was expressed as mg of soluble protein per mg of dry tissue. Before the assay, samples were diluted with AR Assay Buffer to the same concentration. Next, 50 µL of positive control or sample was placed in a well (96-well plate) in duplicate (the second one used for sample background control) and adjusted to 100 µL with 50 µL of AR Assay Buffer, and 60 µL of NADPH solution was added. In the next step, 40 µL of Reaction Mix was added to the samples and positive control, and 40 µL of AR Assay Buffer was added to the sample background control. The absorbance was measured at wavelength λ = 340 nm in kinetic mode for 1 h at 37 °C. Two time points (t1 and t2) in the linear range were chosen for calculating the aldose reductase activity. The experiment was conducted in 3 biological repetitions with *n* ≥ 8. The aldose reductase activity was expressed as nmol NADPH consumed/min/mg of total soluble protein (equivalent to mU/mg protein). For calculation, we used the following formula:

$$\mathrm{Aldose}\;\mathrm{reductase}\;\mathrm{activity}=\frac{B\;\mathrm{sample}-B\;\mathrm{sample}\_\mathrm{background}}{\Delta t\times M}$$

where:

B sample—NADPH amount in sample [nmol];
B sample_background—NADPH amount in sample background [nmol];
Δt—reaction time [min];
M—amount of total soluble protein in reaction solution [mg].

### 5.5. Statistical Analysis

Statistical analysis was performed using GraphPad Prism (version 9) software (Department of Animal Physiology and Developmental Biology AMU licence). Data are presented as individual values with means and standard deviations (SD), where appropriate. Data normality was assessed using the Shapiro–Wilk test, and homogeneity of variances was evaluated using Brown–Forsythe and Bartlett’s tests. For normally distributed data with unequal variances, Welch’s one-way ANOVA followed by the Games–Howell multiple-comparisons test was used. Metabolite data were analyzed according to their distribution and variance characteristics. Normally distributed data with homogeneous variances were analyzed using one-way ANOVA followed by Tukey’s multiple-comparisons test. For normally distributed data with unequal variances, Welch’s ANOVA test was used. Non-normally distributed data were analyzed using the Kruskal–Wallis test followed by Dunn’s multiple-comparisons test. To account for multiple testing across the 12 analyzed metabolite parameters, the Benjamini–Hochberg false discovery rate (FDR) procedure was applied to the *p*-values obtained from the omnibus statistical tests. An FDR-adjusted *p*-value (*q*-value) < 0.05 was considered statistically significant. Percentage changes were calculated relative to the corresponding comparison group, where applicable. For all other analyses, a *p*-value < 0.05 was considered statistically significant.

## Figures and Tables

**Figure 1:**
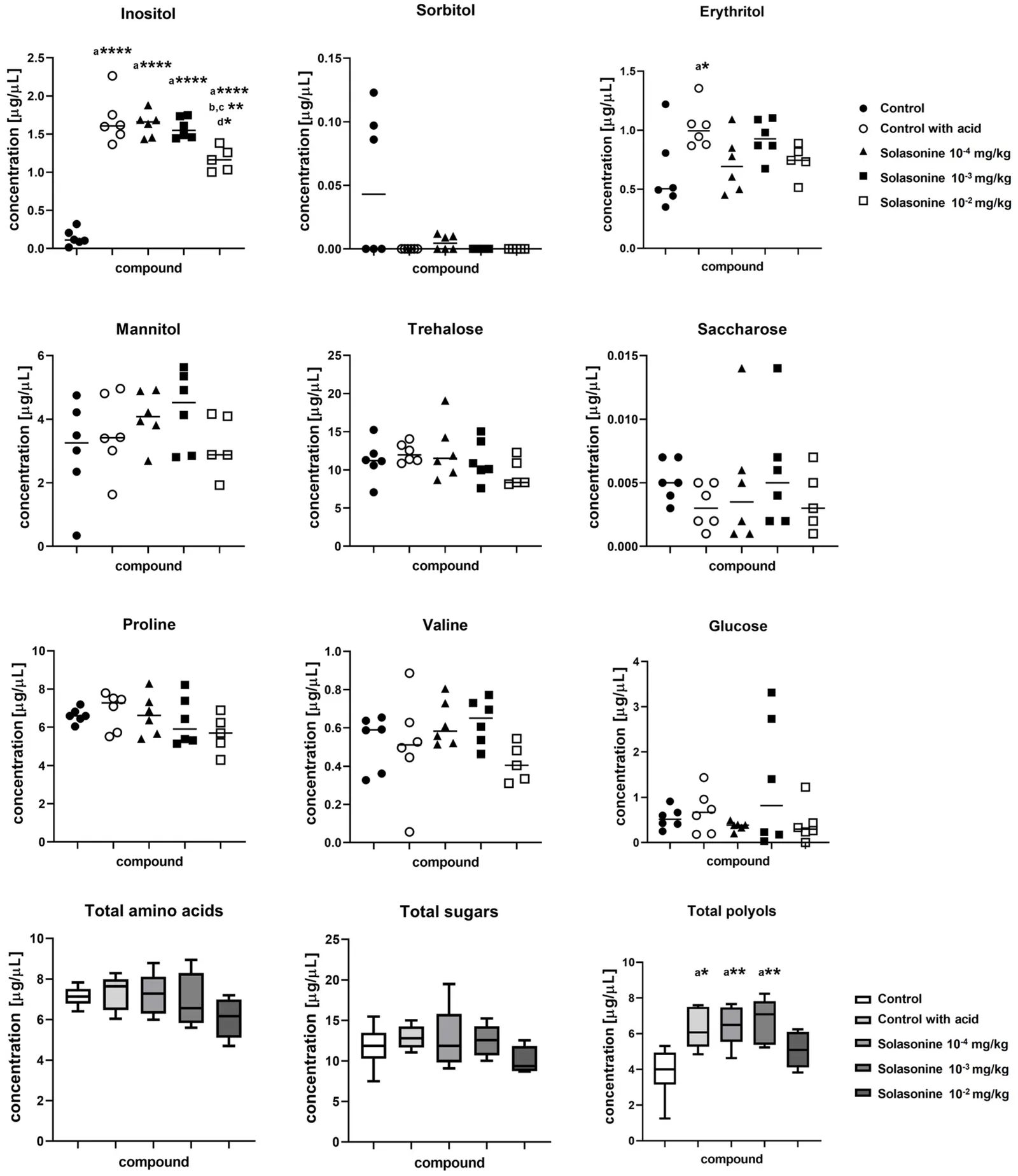
Concentrations of detected compounds determined by RP-HPLC. Statistical significance was assessed using one-way ANOVA followed by Tukey’s multiple-comparisons test for normally distributed data with homogeneous variances, Welch’s ANOVA followed by the Games–Howell multiple-comparisons test for normally distributed data with unequal variances, and the Kruskal–Wallis test followed by Dunn’s multiple-comparisons test for non-normally distributed data. Homogeneity of variances was assessed using the Brown–Forsythe and Bartlett’s tests. * *p* < 0.05, ** *p* < 0.01, **** *p* < 0.0001, compared to ‘a’—control, ‘b’ –control with an acid, ‘c’—solasonine 10^−4^ mg/kg, ‘d’—solasonine in a concentration of 10^−3^ mg/kg.

**Figure 2:**
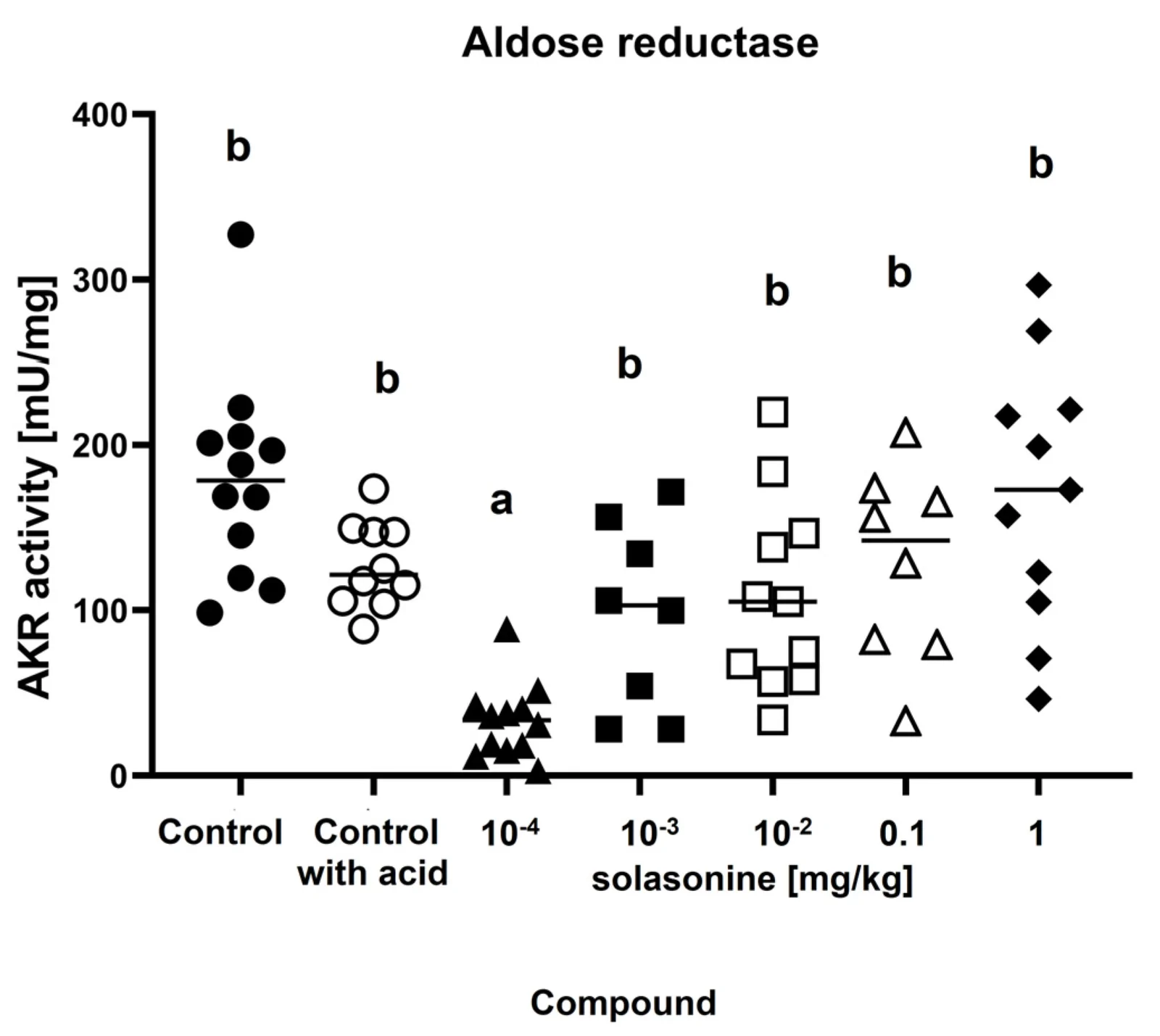
Aldose reductase activity in the fat body of *Galleria mellonella* larvae after exposure to solasonine. Data are presented as individual values with means ± SD. Statistical significance was assessed using Welch’s one-way ANOVA followed by the Games–Howell multiple-comparisons test. Different letters indicate statistically significant differences between groups; groups labeled b differ significantly from the group labeled a (*p* < 0.05).

## Data Availability

The original contributions presented in this study are included in the article/Supplementary Materials. Further inquiries can be directed to the corresponding author.

## Supplementary Materials

The following supporting information can be downloaded at: https://www.mdpi.com/article/10.3390/toxins18100410/s1, Table S1: Supplementary data on metabolite concentrations determined by HPLC and aldose reductase activity in *Galleria mellonella* larvae treated with solasonine.

## Abbreviations

The following abbreviations are used in this manuscript:

AKR	Aldo-keto reductase
RP-HPLC	Reversed-Phase High-Performance Liquid Chromatography
ANOVA	Analysis of variance
